# Structural and Biochemical Characterization of Thioredoxin-2 from *Deinococcus radiodurans*

**DOI:** 10.3390/antiox10111843

**Published:** 2021-11-20

**Authors:** Min-Kyu Kim, Lei Zhao, Soyoung Jeong, Jing Zhang, Jong-Hyun Jung, Ho Seong Seo, Jong-il Choi, Sangyong Lim

**Affiliations:** 1 Radiation Research Division, Korea Atomic Energy Research Institute, Jeongeup 56212, Korea; zl146524@163.com (L.Z.); soyoung@kaeri.re.kr (S.J.); zj10771033@163.com (J.Z.); jungjh83@kaeri.re.kr (J.-H.J.); hoseongseo@kaeri.re.kr (H.S.S.); 2 Interdisciplinary Program for Bioenergy and Biomaterials, Department of Biotechnology and Bioengineering, Chonnam National University, Gwangju 61186, Korea; choiji01@chonnam.ac.kr; 3 Department of Radiation Science and Technology, University of Science and Technology, Daejeon 34113, Korea

**Keywords:** thioredoxin, Trx2, *D. radiodurans*, crystal structure, disulfide reduction

## Abstract

Thioredoxin (Trx), a ubiquitous protein showing disulfide reductase activity, plays critical roles in cellular redox control and oxidative stress response. Trx is a member of the Trx system, comprising Trx, Trx reductase (TrxR), and a cognate reductant (generally reduced nicotinamide adenine dinucleotide phosphate, NADPH). Bacterial Trx1 contains only the Trx-fold domain, in which the active site CXXC motif that is critical for the disulfide reduction activity is located. Bacterial Trx2 contains an N-terminal extension, which forms a zinc-finger domain, including two additional CXXC motifs. The multi-stress resistant bacterium *Deinococcus radiodurans* encodes both Trx1 (DrTrx1) and Trx2 (DrTrx2), which act as members of the enzymatic antioxidant systems. In this study, we constructed Δ*drtrx1* and Δ*drtrx2* mutants and examined their survival rates under H_2_O_2_ treated conditions. Both *drtrx1* and *drtrx2* genes were induced following H_2_O_2_ treatment, and the Δ*drtrx1* and Δ*drtrx2* mutants showed a decrease in resistance toward H_2_O_2_, compared to the wild-type. Native DrTrx1 and DrTrx2 clearly displayed insulin and DTNB reduction activity, whereas mutant DrTrx1 and DrTrx2, which harbors the substitution of conserved cysteine to serine in its active site CXXC motif, showed almost no reduction activity. Mutations in the zinc binding cysteines did not fully eliminate the reduction activities of DrTrx2. Furthermore, we solved the crystal structure of full-length DrTrx2 at 1.96 Å resolution. The N-terminal zinc-finger domain of Trx2 is thought to be involved in Trx-target interaction and, from our DrTrx2 structure, the orientation of the zinc-finger domain of DrTrx2 and its interdomain interaction, between the Trx-fold domain and the zinc-finger domain, is clearly distinguished from those of the other Trx2 structures.

## 1. Introduction

Maintaining a proper redox state in the extracellular or intracellular environments is critical for a variety of cellular processes. The main protein systems that regulate the thiol/disulfide redox balance in the cytoplasm are the thioredoxin (Trx) system, generally composed of thioredoxin reductase (TrxR)/Trx/reduced nicotinamide adenine dinucleotide phosphate (NADPH), and the glutaredoxin (Grx) system, composed of glutathione reductase (GR)/Grx/glutathione (GSH). The Trx system is a ubiquitous redox system found in all living organisms [1], whereas the Grx system is absent in many prokaryotes, including some anaerobic bacteria [2,3]. Interestingly, the Trx systems in several anaerobic organisms utilize reduced nicotinamide adenine dinucleotide (NADH) (e.g., *Thermotoga maritima*, *Desulfovibrio vulgaris*, and *Thermoplasma acidophilum*) [4,5,6], reduced coenzyme F_420_ (F_420_H_2_) (e.g., *Methanocaldococcus jannaschii*) [7,8], or reduced ferredoxin (e.g., *Clostridium pasteurianum*) [9] instead of NADPH.

Trx is a small protein, of approximately 12 kDa, with a highly conserved CXXC motif in its active site [1,10,11,12], which reduces the disulfide bonds in a wide range of proteins. Trx was first identified in *Escherichia coli*. Two cytoplasmic Trx proteins, Trx1 (EcTrx1) [13] and Trx2 (EcTrx2) [14], are found in *E. coli*. EcTrx1 catalyzes the reduction of ribonucleotide reductase (RNR), methionine sulfoxide reductase (Msr), and 3′-phosphoadenylylsulfate (PAPS) reductase, which are important for DNA synthesis, protein repair, and sulfur assimilation, respectively [15,16]. EcTrx2 contains an additional 32 amino acids in its N-terminal region compared to EcTrx1 and shares 28% sequence identity with EcTrx1. EcTrx2 also reduces RNR, the inner membrane protein DsbD [17] and PAPS reductase, similarly to EcTrx1. Although Trx1 is found in all species, some bacteria do not possess Trx2 [15,18,19].

Trx is characterized by a common three-dimensional structure, consisting of four α-helices and five β-sheets [20,21], wherein, its active site CXXC motif, located after the β2-sheet, forms the N-terminal portion of α2 [10,22]. Trx2 contains an additional N-terminal domain, including two CXXC motifs responsible for zinc binding. Based on its structural homology to the zinc-fingers of Npl4 and Vps36, it was proposed that the zinc-finger domain of Trx2 may mediate protein–protein interactions [23]. During the catalytic cycle, the two cysteines in the active site CXXC motif alternately exist in the form of a reduced dithiol or an oxidized disulfide. When Trx reduces a disulfide bridge of its target proteins, it is oxidized and the disulfide in Trx is reduced back to the thiol state by an NADPH-dependent thioredoxin reductase (NTR) [24,25].

The Trx system is important for cellular defense against oxidative stress, because oxidative stress promotes disulfide bond formation in redox-sensitive proteins, thereby functionally modulating these proteins [26]. Furthermore, Trx directly reduces H_2_O_2_ by providing a reduction equivalent to peroxiredoxins (Prxs) [27] and functions as a singlet oxygen quencher, a hydroxyl radical scavenger [28], and a hydrogen donor for peroxidases [29]. Several proteins responsible for oxidative stress response, such as superoxide dismutase, hydrogen peroxidase, ferric uptake regulator, and aconitase, were found to interact with Trx1 in *E. coli* [30]. In addition, alkyl hydroperoxide peroxidase C (AhpC), thiol peroxidase (Tpx), and bacterioferritin comigratory protein (BCP) reportedly also use reduced Trx as an electron donor for the catalytic reduction of substrates [31].

*Deinococcus radiodurans* is extremely resistant to ionizing radiation (IR), ultraviolet (UV), desiccation, and oxidizing agents [32,33,34,35,36]. Cellular exposure to IR increases various reactive oxygen species (ROS) and subsequently causes oxidative damage to DNA, lipids, and proteins [37,38]. *D. radiodurans* has evolved strategies to reduce endogenous ROS production, such as lowering the number of respiratory chain enzymes, importing peptides and amino acids from the environment, and inducing the glyoxylate bypass of the tricarboxylic acid cycle following IR [33,39]. In addition, *D. radiodurans* has efficient enzymatic (catalase, superoxide dismutase, thioredoxin, peroxiredoxin, and peroxidase) and non-enzymatic (deinoxanthin, bacillithiol, pyrroloquinoline-quinone, polyphosphate, and Mn^2+^-metabolite complexes) antioxidant systems that remove the ROS generated by radiation and desiccation [3,33,40].

*D. radiodurans* possesses enzymatic antioxidant systems Trx1 (DrTrx1) and Trx2 (DrTrx2) encoded by *dr0944* and *drA0164*, respectively. Comparative genomic analysis has indicated that Trx1 was present in all 11 analyzed *Deinococcus* species, while Trx2 was only found in three species (*D. radiodurans*, *D. geothermalis,* and *D. deserti*) [35]. It was reported that DrTrx2 has a reduction activity in DrTrxR/NADPH coupled reaction [41]. The crystal structure of DrTrxR was also reported by the same study. When *D. radiodurans* was treated with several TrxR inhibitors, its resistance toward H_2_O_2_ decreased [42]. In this study, we observed the phenotypes of Δ*drtrx1* and Δ*drtrx2* mutants exposed to H_2_O_2_ and measured the reduction activities of native and mutant DrTrx1 and DrTrx2 proteins. Moreover, we solved the crystal structure of DrTrx2 at 1.96 Å resolution, as the first full-length Trx2 structure in a Gram-positive bacterium. A structural comparison with the only two reported full-length Trx2 structures from *Rhodobacter capsulatus* and *Yersinia pestis* revealed that the N-terminal zinc-finger domain orientation of DrTrx2 is rotated 120° counterclockwise, compared to the other Trx2 proteins.

## 2. Materials and Methods

### 2.1. Bacterial Strains and Culture Conditions

*D. radiodurans* strains were grown at 30 °C in TGY broth composed of 0.5% tryptone (Difco Laboratories, Detroit, MI, USA), 0.3% yeast extract (Difco Laboratories, Detroit, MI, USA), and 0.1% glucose (Sigma-Aldrich, St. Louis, MO, USA) or on TGY plates with 1.5% Bacto-agar (Difco Laboratories, Detroit, MI, USA). *E. coli* strains, DH5α (Thermo Fisher Scientific, Waltham, MA, USA) and BL21(DE3) (Novagen, Darmstadt, Germany), were cultivated in Luria-Bertani (LB) broth (Difco Laboratories, Detroit, MI, USA) (1% tryptone, 0.5% yeast extract, 1% NaCl) or on LB medium with 1.5% Bacto-agar at 37 °C. Antibiotics were added to the medium if necessary: ampicillin (Sigma-Aldrich, St. Louis, MO, USA), 100 μg/mL (*E. coli*); kanamycin (Sigma-Aldrich, St. Louis, MO, USA), 50 μg/mL (*E. coli*) and 8 μg/mL (*D. radiodurans*); and chloramphenicol (Sigma-Aldrich, St. Louis, MO, USA), 3 μg/mL (*D. radiodurans*).

### 2.2. Construction of Mutant Strains

Detailed information pertaining to the bacterial strains and plasmids involved in this study are presented in Table S1. Δ*dr0944* (Δ*drtrx1*) and Δ*drA0164* (Δ*drtrx2*) disruption mutants were constructed by targeted mutagenesis using the double crossover recombination method, as previously described [43]. Briefly, PCR amplified 1 kb fragments from the up-stream and down-stream regions of *dr0944* and *drA0164* genes were amplified by polymerase chain reaction (PCR) and subsequently digested using appropriate restriction enzymes and separately ligated to the corresponding sites of pKatAPH3 plasmid. The recombinant plasmids were transformed into *D. radiodurans* cells from exponentially grown cultures. The positive mutant strains harboring a kanamycin resistance marker were selected on TGY agar plates supplemented with 8 μg/mL kanamycin. The disruption of target genes was verified via diagnostic PCR and DNA sequencing. The primers used in this study are listed in Table S2.

Expression plasmids for *drtrx1* and *drtrx2* were constructed using the pRADZ4 vector, which contains the *dr1124* promoter for constitutive gene expression *D. radiodurans* [44]. Complete *drtrx1* and *drtrx2* coding sequences were PCR-amplified from the genomic DNA of *D. radiodurans* using p4_dr0944F/p4_dr0944R and p4_dra0164F/p4_dra0164R primers, respectively, which carry a SpeI restriction site for forward primers and a NotI restriction site for reverse primers (Table S2). The transformed *D. radiodurans* cells were selected using chloramphenicol (3 μg/mL) supplementation.

### 2.3. Quantitative Real-Time Polymerase Chain Reaction (qRT-PCR)

RNA preparation and qRT-PCR were performed as previously described [45]*. D. radiodurans* was cultivated in TGY medium at 30 °C under standard conditions. Cells were grown to log phase (OD_600nm_ ~ 1.0) under normal or stress conditions (20, 40, and 60 mM H_2_O_2_ for 1 h), according to a previous study [43]. Total RNA was purified using an RNeasy Mini kit (Qiagen, Hilden, Germany) and RNase-free DNase (Qiagen, Hilden, Germany), according to the manufacturer’s instructions. For qRT-PCR analysis, cDNA was synthesized from 1 μg of total RNA using a PrimeScript first-strand cDNA Synthesis Kit (Takara, Maebashi, Japan), according to the manufacturer’s instructions. Next, qRT-PCR was performed using SYBR Premix ExTaq (Takara, Maebashi, Japan) on an Eco™ Real-Time PCR System (Illumina, San Diego, CA, USA). The relative expression level of each gene was normalized to *gap*, the gene encoding a glyceraldehyde 3-phosphate dehydrogenase, of *D. radiodurans* as an internal control. Controls to ensure that there was no contaminating genomic DNA in the cDNA (cDNA reaction mixtures generated using RNA with no reverse transcriptase) were also run with each set of qRT-PCR, as previously described [46]. The primers used for qRT-PCR are listed in Table S2.

### 2.4. Phenotype Test of Δdr0944 and ΔdrA0164 Mutants

The sensitivity of *D. radiodurans* to H_2_O_2_ was measured as previously described [43]. Briefly, cells were grown to mid-log phase (OD_600nm_ ~ 1.0) and adjusted to ~10^7^ cfu/mL with fresh TGY medium. These cells were then treated with varying concentrations of H_2_O_2_ for 1 h at 30 °C. Following the above treatments, bovine liver catalase (Sigma-Aldrich, St. Louis, MO, USA) was added in excess (100 g/mL) to eliminate H_2_O_2_ and cells were serially diluted 10-fold (10^−1^ to 10^−6^ fold) in 10 mM phosphate buffer (pH 7.0) and spotted onto TGY plates. Cells were incubated for 3 days at 30 °C prior to colony enumeration. All the data provided here represent the mean and standard deviation of three independent experiments.

### 2.5. Cloning, Expression, and Purification of DrTrxR, DrTrx1, and DrTrx2

The genes *dr1982* (*drtrxR*, GenBank accession No. 1797907), *dr0944* (*drtrx1*, GenBank accession No. 1797257), and *drA0164* (*drtrx2*, GenBank accession No. 1799587) were inserted into the expression plasmid pET-22b (Novagen, Darmstadt, Germany), following which, the resulting constructs expressed residues 2-325 of DrTrxR, 2-109 of DrTrx1, and 2-142 of DrTrx2 protein with an in-frame non-cleavable N-terminal His_6_ tag (MHHHHHH). After verifying the DNA sequence, plasmid DNA was transformed into *E. coli* strain BL21 (DE3) (Stratagene, La Jolla, CA, USA). The cells were grown to an OD_600_ of approximately 0.5 in 1 L of LB medium containing 50 μg/mL of ampicillin (Duchefa, Haarlem, The Netherlands) at 37 °C, and expression was induced with 0.1 mM isopropyl-β-D-thiogalactoside (Duchefa, The Netherlands). Following a 16 h induction at 22 °C, the cells were harvested and resuspended in a final volume of 20 mL of 20 mM Tris-HCl pH 7.5, 200 mM NaCl (buffer A). Then, 2 g of harvested cells were disrupted by sonication using a VP-15s sonicator (Taitec, Koshigaya, Japan). Sonication was performed using the following settings: output power, 8; pulse, 5 s; ON/5 s OFF; temperature, ~2 °C, by floating on ice; and total sonication time, 30 min. Then, cellular debris were discarded via centrifugation at 30,000× *g* for 30 min at 4 °C. The resulting supernatant was loaded onto 5 mL of nickel-nitrilotriacetic acid (Ni-NTA) resin (Qiagen, Hilden, Germany) for gravity-flow affinity chromatography. The resin was washed with 50 mL of buffer A containing 30 mM imidazole. DrTrxR, DrTrx1, and DrTrx2 were eluted with 10 mL of buffer A containing 300 mM imidazole. The partially purified proteins were concentrated to 5 mL and subsequently loaded onto a Superdex 75 HR 16/600 column (GE Healthcare, Boston, MA, USA), pre-equilibrated with buffer A using AKTA-Purifier (GE Healthcare, Boston, MA, USA). Equilibration, protein loading, and elution were performed at a flow rate of 1 mL/min. Finally, purified DrTrxR, DrTrx1, and DrTrx2 were concentrated to approximately 10 mg/mL, 11.6 mg/mL, and 5 mg/mL, respectively, with the volume of around 700 μL for all three proteins.

### 2.6. Crystallization, Data Collection, Data Processing, and Structure Determination

DrTrx2 crystals were grown at 22 °C using the batch crystallization method, with a mother liquor of 0.1M Bis-Tris pH 6.5, 25% *w*/*v* PEG3350. For data collection, DrTrx2 crystals were mounted using a 75 mm MicroMount polymer loop (MiTeGen, Ithaca, NY, USA) and cooled to 100 K using a Cyrostream cooler (Oxford Cryosystems, Oxford, UK). A 1.96 Å resolution native data set was collected at a wavelength of 0.97933 Å using an ADSC Quantum 315r CCD on the beamline BL-7A at the Pohang Light Source, Republic of Korea. Diffraction data were processed and scaled using *DENZO* and *SCALEPACK* from the *HKL-2000* program suite [47]. Molecular replacement phasing using the structure of Trx2 from *R. capsulatus* (PDB ID: 2PPT) [23] as a search model was performed using the *Phaser* program in the *PHENIX* suite [48]. Further model building was completed using *Coot* [49], and refinement was performed with *phenix.refine* in the *PHENIX* suite [48]. All figures showing structures were prepared with PyMOL (https://pymol.org, accessed on 1 February 2021). Data collection and refinement statistics are summarized in Table 1.

### 2.7. Site-Directed Mutagenesis

Site directed mutagenesis was conducted using the pET-22b expression plasmid containing *dr0944* and *drA0164* as a template (Table S2). Amplification was performed using nPfu-Forte DNA polymerase (Enzynomics, Daejeon, Korea). Following temperature cycling, the amplified product was treated with DpnI and transformed into DH5α. The clones were screened on LB plates with ampicillin. Following validation via DNA-sequencing, the mutant plasmids were transformed into BL21(DE3) for expression.

### 2.8. Insulin Reduction Assay

The insulin reduction activities of DrTrx1 and DrTrx2 were measured using DrTrxR and NADPH by monitoring NADPH consumption, which, in turn, was reflected by the decrease in absorbance at 340 nm [50]. The assay mixture (0.5 mL) contained 50 mM Tris-HCl buffer (pH 7.4), 30 mM bovine insulin (Sigma-Aldrich, St. Louis, MO, USA), 0.1 mM NADPH (Sigma-Aldrich), 0.1 μM DrTrxR, and 0.1 μM DrTrx1 or 0.5 μM DrTrx2. All the measurements were carried out at room temperature using an Epoch2 microplate reader (BioTek, Winooski, VT, USA). The reaction was initiated by adding Trx protein, and the resulting change in absorbance was recorded. All the data provided here represent the mean and standard deviation of three independent experiments.

### 2.9. DTNB Reduction Assay

Trx activity was also measured using a 5,5-dithiobis (2-nitrobenzoic acid) (DTNB) reduction assay (Sigma-Aldrich, St. Louis, MO, USA) [14]. The reaction mixture contained 50 mM Tris-HCl buffer (pH 7.4), 0.2 mM NADPH, 0.1 μM DrTrxR, 1.6 mM DTNB (Sigma-Aldrich, St. Louis, MO, USA), and 0.1 μM DrTrx1 or 0.5 μM DrTrx2 in a final volume of 1 mL. The reaction was initiated by adding DTNB and monitored by measuring the increase in absorbance at 412 nm with an Epoch2 microplate reader (BioTek, Winooski, VT, USA). All the data provided here represent the mean and standard deviation of three independent experiments.

## 3. Results and Discussion

### 3.1. Gene Expression and Phenotype Assay under H_2_O_2_ Stress

**Figure 1:**
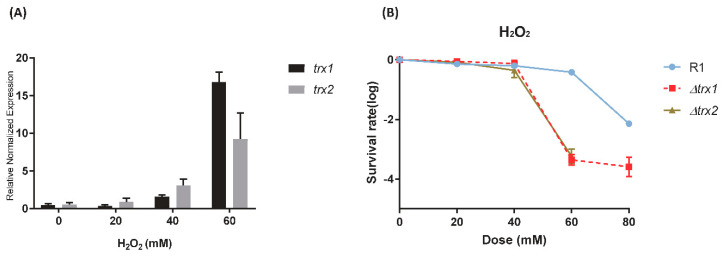
Transcription and phenotype of Δ*drtrx1* and Δ*drtrx2* under H_2_O_2_ stress. (**A**) *D. radiodurans* was exposed to H_2_O_2_ at the indicated concentrations (0, 20, 40, 60 mM), and qRT-PCR analysis was performed to determine *drtrx1* and *drtrx2* transcription level. The fold increase of transcription was determined by setting the expression level of the non-treated control to 1. (**B**) Survival curves of *D. radiodurans* wild-type, Δ*drtrx1*, and Δ*drtrx2* treated with different concentrations of H_2_O_2_ (0, 20, 40, 60, and 80 mM)_._ The cells spotted on TGY plates and colonies were counted after 3 d. Error bars indicate the standard deviation for three experimental replicates.

The *D. radiodurans* genome encodes two Trx proteins, DR0944 (DrTrx1) and DRA0164 (DrTrx2). To evaluate the expression profile of *drtrx1* and *drtrx2*, we performed qRT-PCR, using total RNA isolated from the wild-type *D. radiodurans,* which had been exposed to 20, 40, and 60 mM H_2_O_2_ (Figure 1A). Transcriptional levels of *drtrx1* and *drtrx2* increased more than 2-fold following exposure to 40 mM H_2_O_2_ and almost 17-fold and 9-fold for *drtrx1* and *drtrx2,* following exposure to 60 mM H_2_O_2_ treatment, respectively. To further investigate the roles of DrTrx1 and DrTrx2 in the resistance to oxidative stress, we constructed Δ*drtrx1* and Δ*drtrx2* disruption mutants and examined the resistance of these mutants toward H_2_O_2_. Wild-type Δ*drtrx1*, and Δ*drtrx2* were treated with 20, 40, 60, and 80 mM H_2_O_2_ (Figure 1B). The resistance of ∆*drtrx1* and ∆*drtrx2* towards H_2_O_2_ decreased over a thousand-fold compared to that of the wild-type when the cells were treated with 60 mM of H_2_O_2_ for 1 h. Moreover, ∆*drtrx2* did not survive when the concentration of H_2_O_2_ exceeded 60 mM (Figure 1B), indicating that ∆*drtrx2* was more sensitive to H_2_O_2_ than ∆*drtrx1*. We constructed expression plasmids producing DrTrx1 and DrTrx2 to complement Δ*drtrx1* and Δ*drtrx2*. Complementation by plasmid-borne *drtrx1* and *drtrx2 in trans* showed the restoration of the sensitive phenotype of Δ*drtrx1* and Δ*drtrx2* (Figure S1).

In *E. coli*, it was reported that *trx2* acts as a member of the OxyR regulon and that the expression of *trx2* is induced by H_2_O_2_. In contrast, *trx1* expression is not affected by H_2_O_2_, indicating that *trx1* is not regulated by OxyR. In fact, *trx1* expression in *E. coli* is known to be regulated by guanosine 3′,5′-bispyrophosphate (ppGpp) [16]. The transcription levels of *drtrx1* and *drtrx2* in *D. radiodurans oxyR* mutant (Δ*droxyR*) showed a similar pattern with those of the wild-type after being exposed to 0, 20, and 40 mM H_2_O_2_. Even though the expression of *drtrx1* and *drtrx2* was induced by H_2_O_2_, the defect in *droxyR* did not affect the expression of *drtrx1* and *drtrx2*. This result shows that the mechanisms of the expression regulation of *drtrx1* and *drtrx2* and the correlation between these two genes and *droxyR* may differ from those reported in *E. coli* (Figure S2). However, it should be noted that the DrOxyR is an atypical 1-cysteine, containing OxyR [51], and thus further investigation may be required in order to elucidate the exact role and regulon genes of DrOxyR.

In a previous report, the deletion of *E. coli trx2* resulted in a phenotype that displayed a more sensitive response to H_2_O_2_, whereas deletion of *trx1* or double knock-out of both *trx1* and *trx2* exhibited resistance to even higher levels of H_2_O_2_ [15]. As the cytoplasmic redox potential in these mutants is more oxidized, it is proposed that these highly oxidized conditions activate the oxidative stress response, resulting in the induction of catalase or AhpC, and allowing the mutants to display a higher level of resistance towards H_2_O_2_. Our results, which differed from those pertaining to *E. coli*, showed that, in *D. radiodurans*, the transcription levels of both *trx1* and *trx2* increased with H_2_O_2_ concentrations. In addition, deletion of either *drtrx1* or *drtrx2* resulted in a phenotype that was more sensitive to H_2_O_2_ compared to the wild-type. In Grx-negative bacteria, Trx is known to perform more critical roles in the cellular disulfide balance and oxidative stress response. For instance, *Helicobacter pylori* [52] and *Mycobaterium tuberculosis* [53] lack the Grx-system, and the functional OxyR that regulates the oxidative stress response in these bacteria is different from that of *E. coli*. Since the Grx system does not exist in *D. radiodurans*, the function of Trx proteins and the regulation of *trx* expression appears to differ from those of *E. coli*.

### 3.2. Reduction Activity of DrTrx1 and DrTrx2

**Figure 2:**
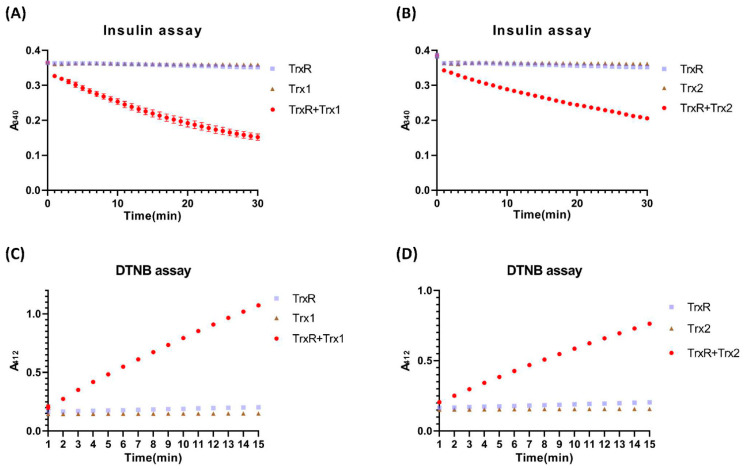
Reduction of insulin and DTNB by DrTrx1 and DrTrx2. Reduction of insulin by DrTrx1 (0.1 μM) (**A**) and DrTrx2 (0.5 μM) (**B**) coupled to the DrTrxR/NADPH regeneration system. Negative controls were represented by the omission of DrTrxR in the presence of each Trx protein or omission of Trx protein in the presence of DrTrxR. Reduction of DTNB by DrTrx1 (0.1 μM) (**C**) and DrTrx2 (0.5 μM) (**D**) coupled to the DrTrxR/NADPH regeneration system. Negative controls are represented by the omission of DrTrxR in the presence of each Trx protein or omission of Trx protein in the presence of DrTrxR. Reduction of insulin (**A**,**B**) was recorded by measuring the decrease in NADPH oxidation at 340 nm. Reduction of DTNB was recorded as an increase in absorption at 412 nm (**C**,**D**). Error bars indicate the standard deviation for three experimental replicates.

To compare the reduction activity of purified DrTrx1 and DrTrx2, we measured the disulfide reduction activity of DrTrx1 and DrTrx2 using an insulin reduction assay. In the presence of DrTrxR/NADPH, both DrTrx1 and DrTrx2 caused a notable decrease in the absorption at 340 nm, indicating that NADPH was consumed during insulin reduction (Figure 2A,B). DrTrx1 showed a relative percentage activity that was 5-fold higher than that of DrTrx2. This result showed that DrTrx1 and DrTrx2 reduce insulin disulfides via the DrTrxR/NADPH regeneration system. Furthermore, the capacity of two Trx proteins to reduce the exposed disulfide bond was explored using DTNB as a generic disulfide substrate. Similarly to the insulin reduction assay, both DrTrx1 and DrTrx2 clearly showed an increase in absorbance at 412 nm, showing that the reduction of DTNB to TNB occurred only in the presence of both DrTrxR and NADPH (Figure 2C,D). The DTNB reduction activity of DrTrx1 was approximately 4-fold higher compared to that of DrTrx2. This result is compatible with a previous report that showed that the catalytic efficiency of Trx1 was three-times higher than that of Trx2 in *B. anthracis* [54].

**Figure 3:**
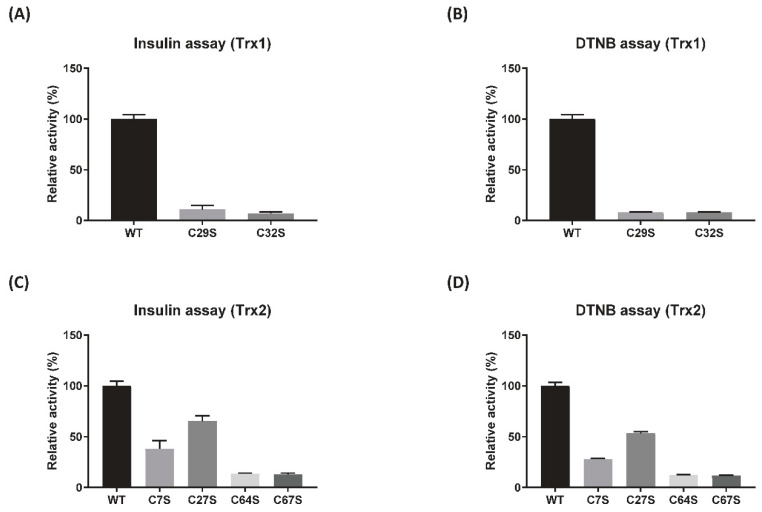
Effects of mutation in the conserved cysteine residues. The reduction activities of DrTrx1, DrTrx2, and its mutant proteins were compared using insulin and DTNB reduction assays coupled to the DrTrxR/NADPH regeneration system. DrTrx1 and its active site CXXC motif mutants (C29S, C32S) were used for insulin (**A**) and DTNB (**B**) reduction assays. DrTrx2 and its zinc-finger domain mutants (C7S, C27S) and active site CXXC motif mutants (C64S, C67S) were used for insulin (**C**) and DTNB (**D**) reduction assays. Relative activities of insulin and DTNB reduction of DrTrx1 and DrTrx2 are depicted as a bar graph, using the absorbance at 15 min (DTNB) and 30 min (insulin), where the activity of native DrTrx1 and DrTrx2 was set to 100%. Error bars indicate the standard deviation for three experimental replicates.

To further investigate the role of cysteines conserved in the CXXC motif of DrTrx1 and DrTrx2, single amino acid substitution mutants, leading to the conversion of Cys to Ser in the CXXC motif were prepared (C29S and C32S in the active site CXXC motif of DrTrx1, C7S and C27S in the N-terminal zinc-binding motif of DrTrx2, and C64S and C67S in the active site CXXC motif of DrTrx2) (Figure S3). Insulin and DTNB reduction assays were performed under the same conditions utilized for native Trx proteins. DrTrx1^C29S^, DrTrx1^C32S^, DrTrx2^C64S^, and DrTrx2^C67S^ mutants completely lost their insulin or DTNB reduction activity, even under conditions that included DrTrxR and NADPH (Figure 3). However, DrTrx2^C7S^ and DrTrx2^C27S^ mutants still showed reduction activity towards insulin and DTNB, with DrTrx2^C27S^ exhibiting a higher activity than DrTrx2^C7S^ (Figure 3C,D and Figure S4). These results suggest that the binding of zinc ion affects the reduction activity of DrTrx2 by stabilizing the N-terminal domain, which is supposedly involved in Trx-target interactions [23]. Additionally, the ratios of molar concentration between DrTrx2 proteins and zinc ion analyzed by inductively coupled plasma optical emission spectrometry (ICP-OES) were 1:1.39, 1:0.63, and 1:0.57 for DrTrx2, DrTrx2^C7S^, and DrTrx2^C27S^, respectively (Table S3). Previous reports have indicated that the zinc binding motif in *E. coli* Trx2 displays an extremely high degree of affinity for zinc ion (*K*_α_ > 10^18^ M^−1^) compared to other reported zinc binding proteins and that formation of the disulfide bond by this zinc binding domain following H_2_O_2_ treatment results in the release of zinc ions and a conformational change [18].

### 3.3. Structural Analysis of DrTrx2

**Figure 4:**
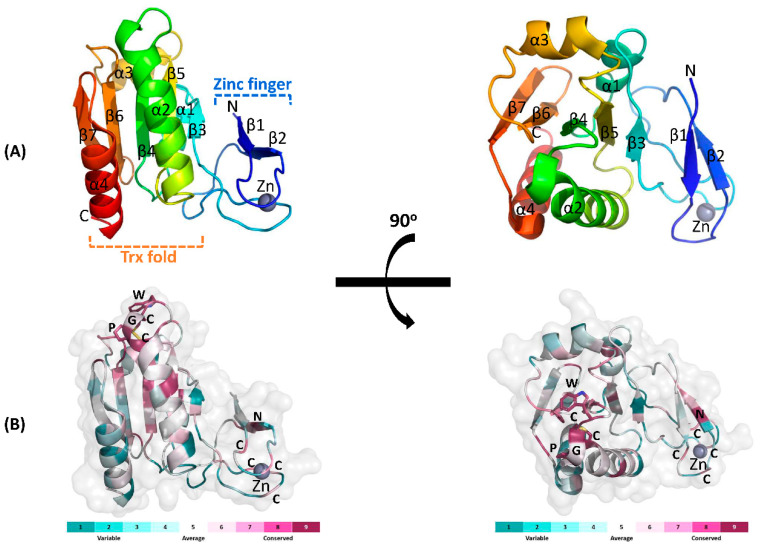
Overall structure of DrTrx2. (**A**) Cartoon representation of the full-length structure of DrTrx2. The molecule is colored progressively, from blue at the N-terminus to red at the C-terminus. The four α-helices (α1-α4) and seven β-sheets (β1–β7) are labelled. One zinc ion bound to the N-terminal zinc-finger domain is shown as a gray sphere. (**B**) Surface conservation of the DrTrx2. The sequence conservation pattern of DrTrx2 was obtained using the Consurf server. Cartoon representation of DrTrx2 shown at the same orientation as (**A**) with a transparent surface. Residues are colored from magenta to cyan with a descending order of conservation, as shown in the color-coding bar below. The active site WCGPC motif, the zinc binding cysteines, and a zinc ion are labelled.

To further investigate the structure–function relationships, we solved the crystal structure of full length DrTrx2 at 1.96 Å resolution. A native data set was collected at a wavelength of 0.97933 Å. DrTrx2 crystal belongs to the *C*2_1_ space group with unit-cell parameters of a = 101.89 Å, b = 57.82 Å, and c = 36.15 Å with one monomer in the asymmetric unit. The DrTrx2 structure was solved by molecular replacement, using the structure of Trx2 from *R. capsulatus* (RcTrx2, PDB ID: 2PPT) [23] as the search model. The electron densities of all residues (Ser2-Ser142) in DrTrx2 were well defined. The final model of DrTrx2 was refined to crystallographic *R* and *R*_free_ values of 19.11% and 22.68%, respectively (Table 1). DrTrx2 is composed of a N-terminal zinc-finger domain and a C-terminal Trx-fold domain. The C-terminal Trx-fold domain is composed of a central β-sheet consisting of five β-strands and four flanking α-helices on either side, where the secondary structural elements are β3-α1-β4-α2-β5-α3-β6-β7-α4. Three β-strands (β3–β5) are parallel, while β6 and β7 are antiparallel. The N-terminal zinc-finger domain, coordinated with one zinc ion, consists of two β-sheets (β1 and β2) connected by four loops and is linked to β3 in the C-terminal Trx-fold domain (Figure 4A). The amino acid sequence of DrTrx2 was used to compare against 150 selected homologous sequences using the ConSurf server (https://consurf.tau.ac.il/, accessed on 17 April 2021) [55], to analyze the surface conservation map (Figure 4B). The results showed that the active site residues ^63^WCGPC^67^ in the Trx-fold domain are highly conserved and that the cysteine residues of two zinc binding CXXC motifs are also conserved at a relatively high rate, along with several conserved residues in β2 of the N-terminal domain.

**Figure 5:**
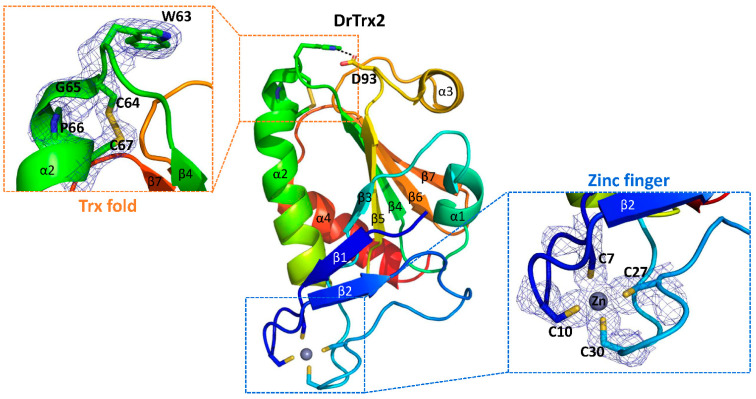
Conserved cysteine residues in DrTrx2. A cartoon representation of the full-length structure of DrTrx2 is provided in a different orientation compared to Figure 4. The molecule is colored progressively, from blue at the N-terminus to red at the C-terminus. The amino acid residues in the active site ^63^WCGPC^67^ motif, the N-terminal zinc binding site, and Asp93, which makes a hydrogen bond (dotted black line) with Trp63, are shown as sticks. Dotted rectangles indicate enlarged views of the active site ^63^WCGPC^67^ motif (orange) and the N-terminal zinc binding site (blue). The zinc ion is shown as a gray sphere. The final maximum-likelihood weighted 2*F*_o_-*F*_c_ electron density map of ^63^WCGPC^67^ and four cysteine residues coordinated with zinc ion contoured at 1σ are also presented as mesh.

The active site ^63^WCGPC^67^ motif is located at the solvent accessible loop following β2 and at the beginning of α2. In our DrTrx2 structure, in its oxidized state, two cysteines form a disulfide bond with a clearly connected electron density map (Figure 5). Trp63 makes a hydrogen bond with Asp93 located in the loop between β5 and α3. This hydrogen bond was presumably participated in the stabilization of the active site configuration [56], and the same hydrogen bonds were also observed in Trx proteins from bacteria, such as *E. coli* (PDB ID: 2TRX) [22], *Staphylococcus aureus* (PDB ID: 2O7K) [57], and *Mycobacterium tuberculosis* (PDB ID: 2I1U) [58]. The side chain of Arg68 in DrTrx2 directs towards the solvent region, while the corresponding Arg77 of RcTrx2 interacts with the backbone carbonyl group of proline residue, which precedes the conserved tryptophan residue in the WCGPC motif [23]. In the N-terminal zinc-finger domain, two CXXC motifs are located in the loops at the end of β-strands. Four cysteine residues form a tetragonal zinc-binding site and coordinated with one zinc ion (Figure 5). The zinc ion may participate in the association of β1, β2, and connecting loops, along with hydrophobic interactions, which are mainly formed by Leu5 in β1, Val16 in β2, and Pro25 and Leu34 in the connecting loops, to maintain the correct structural conformation of the N-terminal zinc-finger domain. Although this zinc binding site is apart from the active site, the DrTrx2^C7S^ and DrTrx2^C27S^ mutants showed decreased reduction activity (Figure 3), indicating that the proper conformation of the N-terminal zinc-binding domain also affects the complete enzymatic activity of Trx2 protein.

### 3.4. Structural Comparison with Other Trx2 Proteins

**Figure 6:**
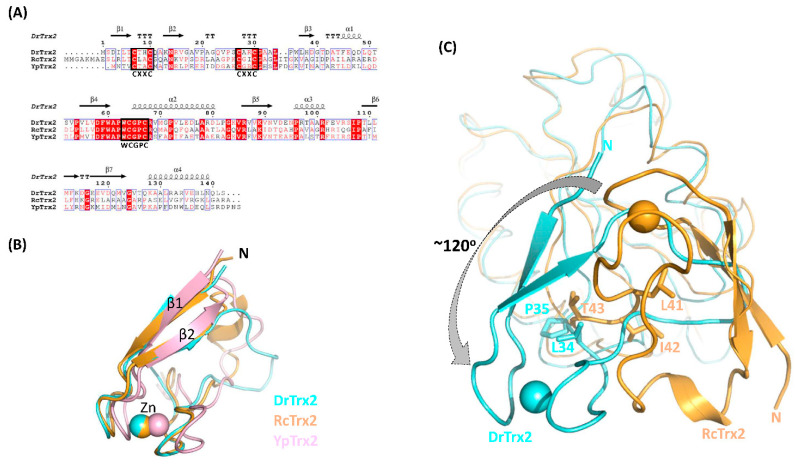
Structural comparison of DrTrx2 with other Trx2 proteins. (**A**) The structure-based sequence alignment of DrTrx2 with RcTrx2 and YpTrx2. The programs, Multalign (http://multalin.toulouse.inra.fr, accessed on 6 March 2021) and Espript (http://espript.ibcp.fr (accessed on 6 March 2021)), were used to visualize the alignment. White letters on red shading represent 100% identity. (**B**) Superimposition of the structures of the N-terminal zinc-finger domains from DrTrx2 (cyan), RcTrx2 (orange), and YpTrx2 (pink). The bound zinc ion for each protein is shown as a sphere. (**C**) Superimposition of the overall structures of DrTrx2 (cyan) and RcTrx2 (orange). For clarity, only DrTrx2 and RcTrx2 structures are shown. The C-terminal Trx-fold domains are shown as a ribbon diagram and the N-terminal zinc-finger domains are represented in cartoon. Lys43 and Pro35 from DrTrx2 and Leu41, Ile42, and Thr43 from RcTrx2 are shown in sticks. The bound zinc ion for each protein is shown as a sphere, and the difference between the domain orientations of two proteins is marked with an arrow.

To our knowledge, only two full-length Trx2 structures have been deposited in the PDB database so far; one from *R. capsulatus* (RcTrx2, PDB ID: 2PPT) as a reduced form [23] and the other from *Yersinia pestis* (YpTrx2, PDB ID: 3P2A, unpublished) as an oxidized form. RcTrx2 and YpTrx2 are composed of 152 and 145 amino acids and show 37% and 41% of sequence identity with DrTrx2, respectively. Multiple sequence alignment clearly showed that six cysteine residues in the N-terminal zinc binding CXXC motifs and the active site CXXC motif are well conserved among DrTrx2, RcTrx2, and YpTrx2 (Figure 6A).

To compare the overall structures of the three Trx2 proteins, structures of RcTrx2 and YpTrx2 were superimposed onto that of DrTrx2. As well defined in many studies, the C-terminal Trx-fold domain of DrTrx2 is almost identical with the Trx-fold domain of RcTrx2 and YpTrx2, with r.m.s.d. values of 1.2 Å for 77 C^α^ atoms and 0.9 Å of 87 C^α^ atoms, respectively (Figure S5). The N-terminal zinc-finger domain of DrTrx2 also shares a similar structure with those of RcTrx2 and YpTrx2, with r.m.s.d. values of 0.5 Å for 24 C^α^ atoms and 0.4 Å for 22 C^α^ atoms, respectively, except for the additional 3_10_-helix (RcTrx2) or loop orientation (YpTrx2) followed by β2 (Figure 6B). While the overall structures of RcTrx2 and YpTrx2 are almost identical, with an r.m.s.d. value of 1.5 Å for 123 C^α^ atoms, we found highly notable differences between the orientation of the zinc-finger domains of DrTrx2 of the three Trx2 proteins, compared to those of RcTrx2 and YpTrx2. The entire N-terminal zinc-finger domain of DrTrx2 exhibited an approximately 120° counterclockwise rotation compared to the position of the RcTrx2 zinc-finger domain (Figure 6C). The difference between the orientations of the N-terminal domains of the two proteins occurred at the Pro35 residue in DrTrx2. Although the adjacent Leu34 is also conserved in RcTrx2 (Leu41), two additional amino acids (Ile42, Thr43) are inserted between Leu41 and Gly44 (corresponding to Pro35 in DrTrx2) in RcTrx2. This insertion may force the N-terminal domain of RcTrx2 close to the Trx-fold domain, while Pro35 of DrTrx2 impose a twist in the relative orientation of its zinc-finger domain, by the restricted flexibility of the proline peptide bond.

The different orientations of the N-terminal zinc-finger domain seen among these proteins also affected the interaction between N- and C-terminal domains of each protein. In RcTrx2, Arg11, and Thr13 from β1 make hydrogen bonds with Arg56 in α1, Asp60, and Asp61 in the loop between α1 and β3, and Arg95 in β4. In addition, Gly18 and Leu41 in the N-terminal domain make hydrogen bonds with the side chains of Arg56 and Arg95, respectively. YpTrx2 shows similar inter-domain interactions with RcTrx2. In YpTrx2, Thr3, Asp51, Asp52, and Arg86 are equivalent to the Thr13, Asp60, Asp61, and Arg95 of RcTrx2, in forming intensive hydrogen bonds, while there is no residue equivalent to RcTrx2, Arg11, and Arg56. Instead, Arg18 in the N-terminal domain interacts with the backbone oxygen atom of Gly83 in YpTrx2. Notably, DrTrx2 differs from the other two Trx2 proteins, in that DrTrx2 does not display a clear hydrogen bond between N- and C-terminal domains, except for water-mediated polar interactions between Asp3 and Asp39 (Figure S6).

The N-terminal zinc-finger domain of bacterial Trx2 proteins is thought to be involved in the interaction between Trx2 and its target. Our preliminary results for the identification of DrTrx2 targets showed that DrTrx2 might interact with thiosulfate sulfurtransferase (TST), which is known to be a target of human Trx (data not shown). In contrast, DrTrx2 did not show clear interactions with DnaK or DnaJ, which were reported to interact with EcTrx2 and RcTrx2 [23,59]. The different orientation of DrTrx2 N-terminal zinc-finger domain compared to RcTrx2 and YpTrx2 might cause, not only the distinct interdomain interactions, but also differences in the surface exposed region that are important for protein–protein interactions, and thus may be implicated in the recognition of the unique targets of DrTrx2. Further investigation of the structural flexibility of the zinc-finger domain of DrTrx2 and its effect on the function and target recognition of DrTrx2 may yield important evidence regarding the role of Trx in many vital cellular processes.

## 4. Conclusions

Since the first three-dimensional structure of Trx in *E. coli* was reported, many Trx structures, in a wide range of species, have been reported. Structural analyses of Trx proteins have revealed the detailed mechanism underlying thiol/disulfide exchange, as well as the conserved amino acids responsible for maintaining the active site in its functional state, and even the evolutionary diversity that exists in this ubiquitous protein family. Although the number of identified Trx2 in bacteria is growing continuously and despite Trx2 being known to conduct important roles in the regulation of the cellular redox state and oxidative stress response in bacteria, information regarding the structure and function of bacterial Trx2 has been much less than that for Trx1, until now. Here, we present atomic level details of the structure of full-length DrTrx2. The most significant difference between it and the other two reported full-length Trx2 structures is the orientation of the N-terminal zinc-finger domain, which is known to be involved in protein–protein interactions. The reduction activity of DrTrx2 was decreased by defective zinc binding, but not fully eliminated. Unlike in *E. coli*, the expression levels of *drtrx1* and *drtrx2* were increased and the deletion of these genes produced a sensitive phenotype under exposure to H_2_O_2_. Considered together, our recent structural and functional characterizations of Trx proteins from multi-stress resistant *D. radiodurans* may enhance our knowledge regarding the cellular function of bacterial Trx2 proteins.

## Figures and Tables

**Table 1:** Data collection and refinement statistics.

**Data Collection**	
Data set	DrTrx2
PDB ID	7D6L
Diffraction source	PAL BL-7A
Wavelength (Å)	0.97933
Space group	*C*2_1_
*a, b, c* (Å)	101.89, 57.82, 36.15
α, β, γ (°)	90, 94.78, 90
Resolution range (Å) *	50.00–1.96 (1.99–1.96)
No. of unique reflections	14566 (685)
Completeness (%) *	94.9 (91.3)
Redundancy *	5.1 (3.9)
*I/σ(I)* *	50.78 (11.63)
*R*_sym_ (%) *^,†^	6.5 (23.2)
**Refinement**	
Resolution range (Å)	18.96–1.95
No. reflections	14526
No. atoms	
Protein	1087
Water	74
Zinc	1
*B*-factors	
Protein	37.71
Water	48.57
Zinc	25.68
*R* (*R*_free_) (%) *, ^‡^	19.11 (22.68)
R.m.s. deviations ^§^	
Bonds length (Å)	0.018
Bond Angles (°)	1.126

* The numbers in parentheses are for the outer shell. ^†^ *R*_sym_ = Σ*_hkl_* Σ*_i_* |*I_i_*(*hkl*) - <I(*hkl*)> |/Σ*_hkl_* Σ*_I_ I_i_*(*hkl*), where *I_i_*(*hkl*) is the intensity of the observed reflection *hkl* and <I(*hkl*)> is the mean intensity of symmetry-equivalent reflections. ^‡^ *R* = Σ |*F*_o_-*F*_c_|/Σ *F*_o_, where *F*_o_ is the observed structure factors and *F*_c_ is the structure factors calculated from the atomic model. *R*_free_ was calculated with 10% of the reflections. ^§^ R.m.s. deviations in bond length and angles are the deviations from ideal values.

## Data Availability

The atomic coordinates and structure factors for DrTrx2 have been deposited in the Protein Data Bank, with the accession code 7D6L. Other data is contained within this article and Supplementary File.

## Supplementary Materials

The following are available online at https://www.mdpi.com/article/10.3390/antiox10111843/s1, Figure S1: Complementation of Δ*drtrx1* and Δ*drtrx2*, Figure S2: Transcription of *drtrx1* and *drtrx2* in Δ*droxyR* mutant under H_2_O_2_ stress, Figure S3: The structure-based sequence alignment of DrTrx2 with DrTrx1, Figure S4: Reduction of insulin and DTNB by cysteine mutant Trx proteins, Figure S5: Structural comparison of DrTrx2, RcTrx2, and YpTrx2, Figure S6: Interdomain interactions, Table S1: Bacterial strains and plasmids used in this study, Table S2. Primers used for gene cloning, Table S3. Zinc concentrations measured by ICP-OES.
