# Supplementary material for: Structural and Biochemical Characterization of Thioredoxin-2 from Deinococcus radiodurans
Source: Antioxidants (Basel). 2021 Nov 20;10(11):1843. doi: 10.3390/antiox10111843 (PMC8615215; doi:10.3390/antiox10111843)
Supplement: Supplementary file 1 [file antioxidants-10-01843-s001.zip › antioxidants-1464755-supplementary.pdf]

Supplementary Information

1. Supplementary Figures

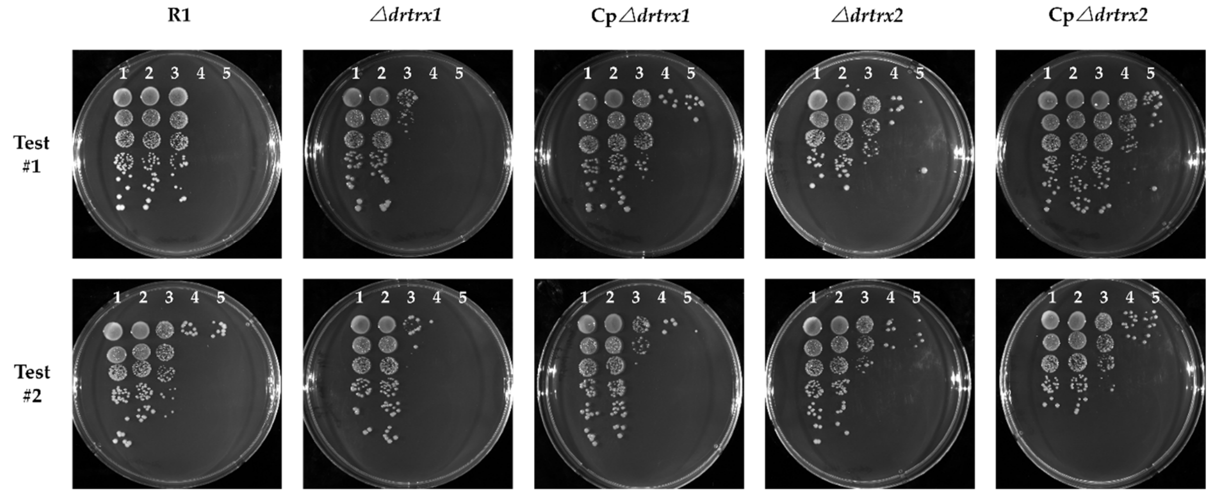

Figure S1. Complementation of Δ*drtrx1* and Δ*drtrx2*. *D. radiodurans* wild-type, Δ*drtrx1* harboring the *drtrx1* expression plasmid (CpΔ*drtrx1*), and Δ*drtrx2* harboring the *drtrx2* expression plasmid (CpΔ*drtrx2*) were spotted on TGY plates supplemented with 0 (1), 20 (2), 40 (3), 60 (4), and 80 (5) mM H_2O_2. The plates were incubated for 2 days at 30 °C prior to the enumeration of colonies.

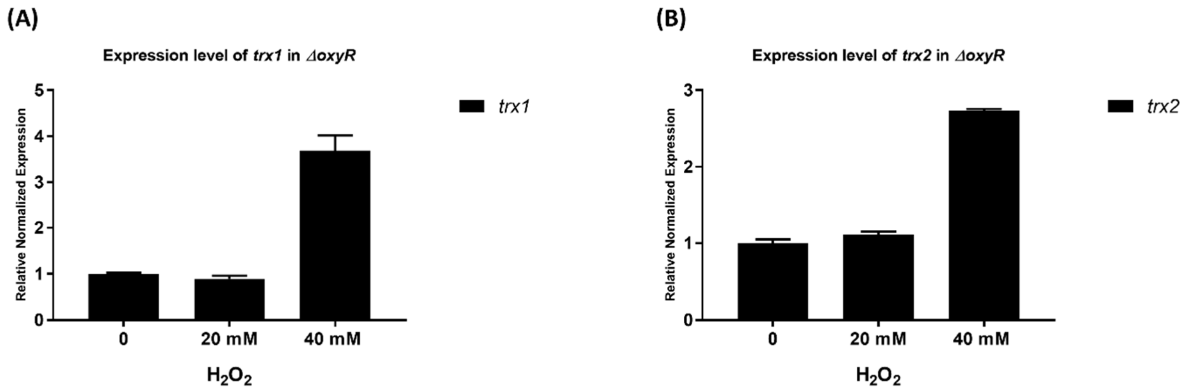

Figure S2. Transcription of *drtrx1* and *drtrx2* in Δ*droxyR* mutant under H_2O_2 stress. *D. radiodurans* Δ*droxyR* mutant was exposed to 0, 20, and 40 mM H_2O_2, then qRT-PCR analysis was performed to determine *drtrx1* (**A**) and *drtrx2* (**B**) transcription level. The fold increase of transcription was determined by setting the expression level of the non-treated control as 1. Error bars indicate the standard deviation for three experimental replicates.

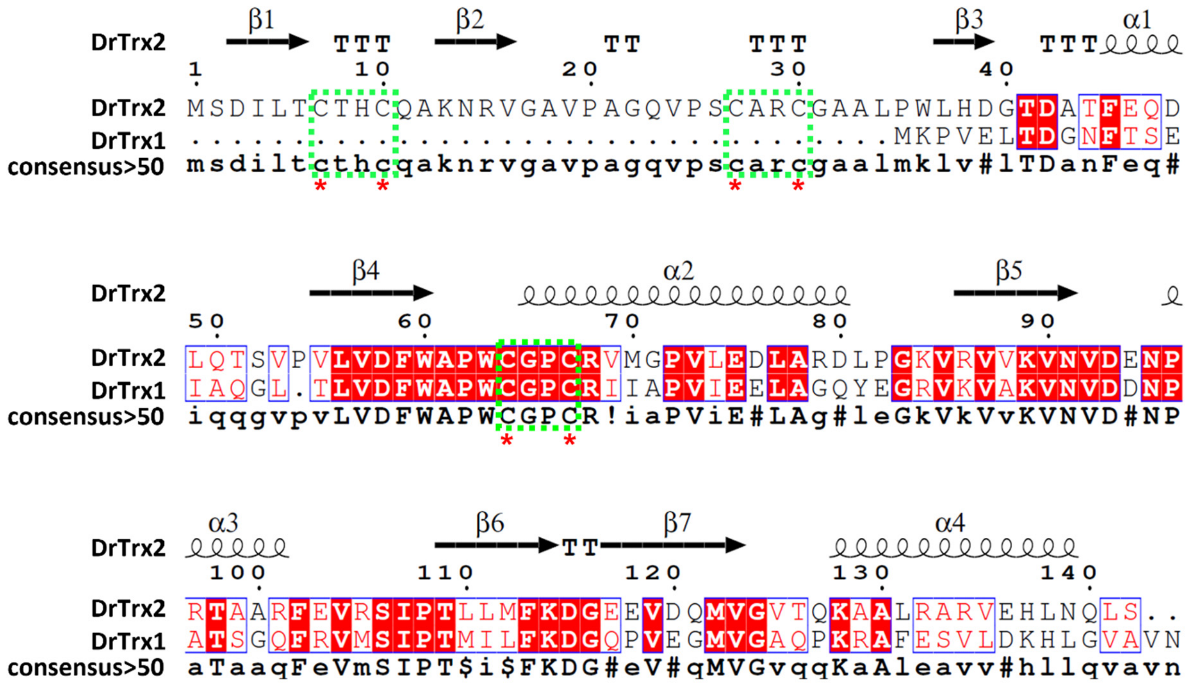

Figure S3. The structure-based sequence alignment of DrTrx2 with DrTrx1. The programs, Multalign (http://multalin.toulouse.inra.fr) and Espript (http://espript.ibcp.fr), were used to visualize the alignment. White letters on red shading represent 100% identity. Cysteine residues in the active site CXXC motif and the zinc-binding domain of DrTrx2 are marked with green dotted rectangles and red asterisks.

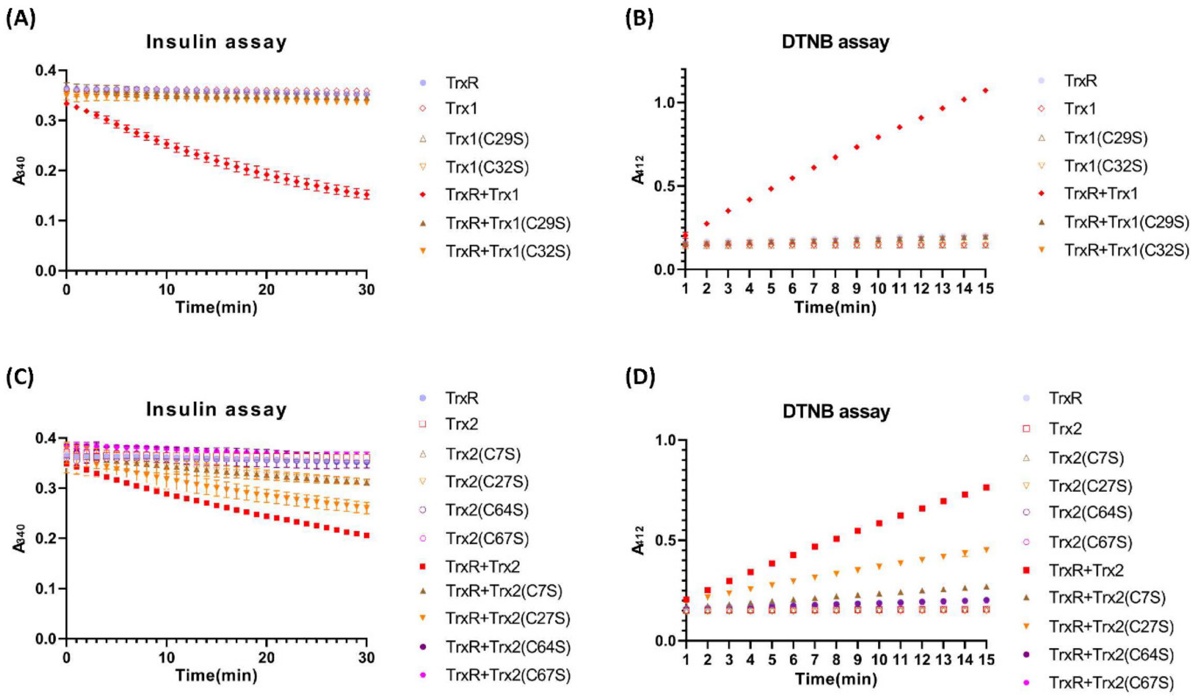

Figure S4. Reduction of insulin and DTNB by cysteine mutant Trx proteins. Reduction of insulin by DrTrx1, DrTrx1^{C29S}, and DrTrx1^{C32S} (0.1 μM) (**A**) and DrTrx2, DrTrx2^{C7S}, DrTrx2^{C27S}, DrTrx2^{C64S}, and DrTrx2^{C67S} (0.5 μM) (**C**) coupled to the DrTrxR/NADPH regeneration system. Negative controls are the omission of DrTrxR in the presence of each Trx protein or omission of Trx protein in the presence of DrTrxR. Reduction of DTNB by DrTrx1, DrTrx1^{C29S}, and DrTrx1^{C32S} (0.1 μM) (**B**) and DrTrx2, DrTrx2^{C7S}, DrTrx2^{C27S}, DrTrx2^{C64S}, and DrTrx2^{C67S} (0.5 μM) (**D**) coupled to the DrTrxR/NADPH regeneration system. Negative controls are the omission of DrTrxR in the presence of each Trx protein or omission of Trx protein in the presence of DrTrxR. The reduction of insulin (**A, C**) was recorded by measuring the decrease of NADPH oxidation at 340 nm. The reduction of DTNB was recorded as an increase in absorption at 412 nm (**B, D**). Error bars indicate the standard deviation for three experimental replicates.

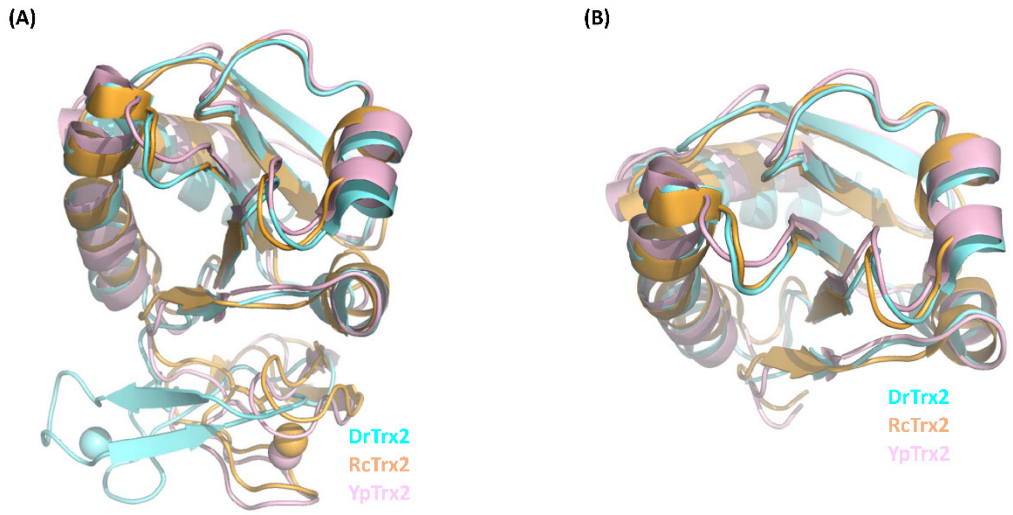

Figure S5. Structural comparison of DrTrx2, RcTrx2, and YpTrx2. Superimposition of the overall structures of DrTrx2, RcTrx2, and YpTrx2 (**A**) and the C-terminal Trx-fold domains of DrTrx2, RcTrx2, and YpTrx2 (**B**). Structures are shown as cartoon representation and the bound zinc ions are shown as sphere.

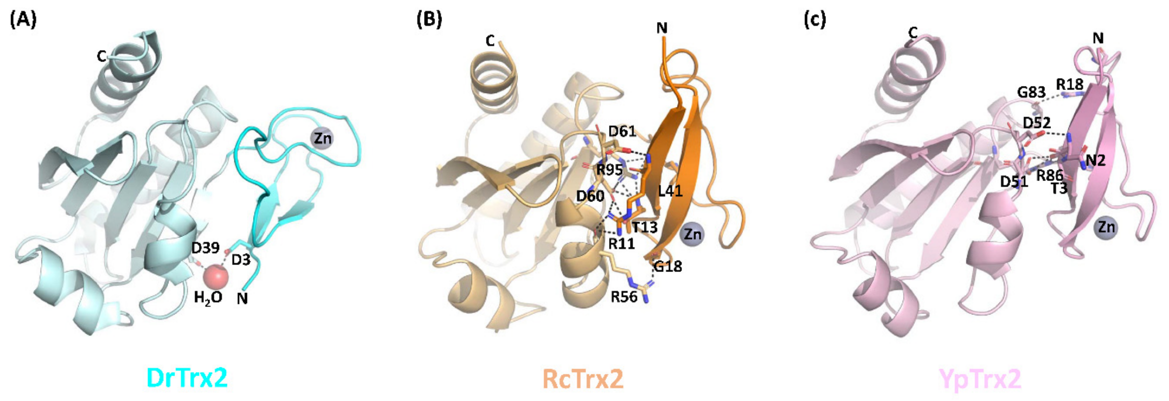

Figure S6. Interdomain interactions. Residues involved in the interdomain hydrogen network of DrTrx2 (**A**), RcTrx2 (**B**), and YpTrx2 (**C**) are shown as sticks and the hydrogen bonds are indicated as dotted black lines. Zinc ions and water molecule are represented as spheres. Overall structures are indicated as cartoon representation. Different colour intensity is used to distinguish the N- (weak) and C-terminal (strong) domain of Trx2.

2. Supplementary tables

Table S1. Bacterial strains and plasmids used in this study

Strain or plasmid	Description	Reference
**Strains**		
D. radiodurans		
R1	wild-type (ATCC13939)	Lab stock
Δtrx1	*trx1* null mutant, Km^r	This study
Δtrx2	*trx2* null mutant, Km^r	This study
E. coli		
DH5*α*	Host for plasmid subclones	Lab stock
BL21 (DE3)	Host for protein expression	Lab stock
**Plasmids**		
pET22b	IPTG-inducible expression vector with N-terminal Histag, Amp^r	This study
pET-TrxR	pET22b a derivative for expression of *trxR*	This study
pET-Trx1	pET22b a derivative for expression of *trx1*	This study
pET-Trx2	pET22b a derivative for expression of *trx2*	This study
pET-Trx1 (C29S)	pET22b a derivative for expression of *trx1 (C29S)*	This study
pET-Trx1 (C32S)	pET22b a derivative for expression of *trx1 (C32S)*	This study
pET-Trx2 (C7S)	pET22b a derivative for expression of *trx2 (C7S)*	This study
pET-Trx2 (C27S)	pET22b a derivative for expression of *trx2 (C27S)*	This study
pET-Trx2 (C7S, C27S)	pET22b a derivative for expression of *trx2 (C7S, C27S)*	This study
pET-Trx2 (C64S)	pET22b a derivative for expression of *trx2 (C64S)*	This study
pET-Trx2 (C67S)	pET22b a derivative for expression of *trx2 (C67S)*	This study
pRD4-Trx1	pRADZ4 a derivative for expression of *trx1*	This study
pRD4-Trx2	pRADZ4 a derivative for expression of *trx2*	This study
pkatAPH3	PUC19 containing *aph* and *D. radiodurans katA* promoter; 2.3 kb; Km^r	Lab stock
pRADZ4	*E. coli-D. radiodurans* shuttle vector carrying *D. radiodurans groES promotor regulated LacZ and promotor of dr1124; 10 kb;* Amp^r in *E. coli* and Cm^r in *D. radiodurans*	Lab stock

Table S2. Primers used for gene cloning.

Primer	Sequence (5′ to 3′)	Description
Kan-diaF	TACCTGCGCCGGTTGCATTCGATTCC	Diagnostic PCR
Kan-diaR	TAGGAATCGAATGCAACCGGCGCAGG	
dr0944 upF	TACTCGAGCATCGTCATCATGAC (*Xho* I)	Construction of pKatAPH3-*dr0944*
dr0944 upR	TAGATATCGAACCCCGAATTGCG (*EcoR* V)	
dr0944 downF	TAGGATCCTTTTCAAGCCCTCAT (*BamH* Ⅰ)	
dr0944 downR	TAGCATGCGCGGTGGATATGGCG (*Sph* Ⅰ)	
dr0944 diaF	TAGGTGCGTGAGCTGCGGCA	
dr0944 diaR	TACCGGCATTCCCCCGCACA	
dra0164 upF	TACTCGAGATCGG CTGACGGTGCCG (*Xho* I)	Construction of pKatAPH3-*dra0164*
dra0164 upR	TAGATATCGGGGTCCTCCGGGGGAAA (*EcoR* V)	
dra0164 downF	TAGGATCCACGGCGCTGCTTCGCTGG (*BamH* Ⅰ)	
dra0164 downR	TAGCATGCGTGCTGGGTGCTGGCGGT (*Sph* Ⅰ)	
dra0164 diaF	TAATGACTGACCTGCTCGAC	
dra0164 diaR	TAAACTGCCGGGCATACTCC	
p4_dr0944F	TAACTAGTATGCGGGCCAGCATACCC (*Spe* I)	pRADZ4-*dr0944*
p4_dr0944R	TAGCGGCCGCTCAGTTGACAGCGACCC GAG (*Not* I)	
p4_dra0164F	TAACTAGTATGAGTGACATCCTGACC (*Spe* I)	pRADZ4-*dra0164*
p4_dra0164R	TAGCGGCCGCTCAGGAAAGCTGGTTGAGGTG (*Not* I)	
*dr1982*F	TACATATGCATCATCATCATCATCATACGGCACCTAC TGCACAC (*NdeI*)	pET22b-*dr1982*
*dr1982*R	TACTCGAGTCAGTCGGCAGCCGTGACCTC (*XhoI*)	
*dr0944*F	TACATATGCATCATCATCATCATCATAAGCCTGTGGA CTCACG (*Nde* I)	pET22b-*dr0944*
*dr0944*R	TACTCGAGTCAGTTGA AGCGACCCC (*Xho* I)	
*dra0164*F	TACATATGCATCATCATCATCATCATAGTGACATCCT GACCTGT (*Nde* I)	pET22b-*dra0164*
*dra0164*R	TACTCGAGTCAGGAAAGCTGGTTGAGGTG (*Xho* I)	
*dr0944*_C29S F	GGGCGCCCTGGAGTGGCCCTTGC	Site-directed mutagenesis of *dr0944* (C29S)
*dr0944*_C29S R	GCAAGGGCCACTCCAGGGCGCCC	
*dr0944*_C32S F	CCCTGGTGTGGCCCTAGCCGCATC	Site-directed mutagenesis of *dr0944* (C32S)
*dr0944*_C32S R	GATGCGGCTAGGGCCACACCAGGG	
*dra0164*_C7S F	GTGACATCCTGACCAGTACCCACTGCCAG	Site-directed mutagenesis of *dra0164* (C7S)
*dra0164*_C7S R	CTGGCAGTGGGTACTGGTCAGGATGTCAC	
*dra0164*_C27S F	AGGTGCCGAGCAGCGCCCGCTGC	Site-directed

*dra0164*_C27S R	GCAGCGGGCGCTGCTCGGCACCT	mutagenesis of *dra0164*(C27S)
*dra0164*_C64S F	GGCGCCGTGGAGCGGCCCCTG	Site-directed mutagenesis of *dra0164*(C64S)
*dra0164*_C64S R	CAGGGGCCGCTCCACGGCGCC	
*dra0164*_C67S F	GGTGCGGCCCCAGCCGCGTGATG	Site-directed mutagenesis of *dra0164*(C67S)
*dra0164*_C67S R	CATCACGCGGCTGGGGCCGCACC	
dr0944 TF	TGACCCTGGTCGACTTCTGG	RT-PCR
dr0944 TR	CTTGACGCGGCCTTCGTACT	
dra0164 TF	GTCAACGTGGACGAGAACCC	RT-PCR
dra0164 TR	CCATCTGGTCCACCTCTTCCC	

Table S3. Zinc concentrations measured by ICP-OES

Sample	Trx concentration (mg/ml)	Trx concentration (μM)	Element/ Wavelength(nm)	Zn concentration (mg/L)	Zn concentration (μM)	Trx:Zn ratio
DrTrx2	0.2	12.87	Zn/206.2	1.17	17.89	1:1.39
DrTrx2 C7S	0.2	12.87	Zn/206.2	0.53	8.11	1:0.63
DrTrx2 C27S	0.2	12.87	Zn/206.2	0.48	7.34	1:0.57
